# Psychometric evaluation of the Chinese version of self-assessment scale for the community- based and emergency practice among medical students

**DOI:** 10.3389/fpubh.2025.1673319

**Published:** 2025-09-29

**Authors:** Qi Hu, Chen Zheng, Xue Yang

**Affiliations:** ^1^The First Affiliated Hospital of Jinzhou Medical University, Jinzhou, Liaoning, China; ^2^Department of Nursing, Shanxi Bethune Hospital, Taiyuan, Shanxi, China

**Keywords:** community medicine, emergency practice, self-assessment, psychometric assessment, factor analysis

## Abstract

**Objective:**

As the population continues to age, community-based primary services and treatment for older people have become the most important part of the public health system. The aim of this study was to translate the Self-assessment scale for the community- based and emergency practice (C-CEP) into Chinese and to test its reliability and validity among medical students.

**Methods:**

After gaining access to the C-CEP scale, the Brislin translation-back translation model was used for translation and cross-cultural adaptation. 600 medical students were recruited to participate in this study using a convenience sampling method (*n* = 600). General information about the study population was analyzed by descriptive statistics; item analysis was used to screen the scale items; the reliability of the Chinese version of the questionnaire was measured by internal consistency, split-half reliability, and retest reliability; the validity of the questionnaire was measured by content validity and structural validity.

**Results:**

The English version of the C-CEP consisted of 15 items and the analysis of the items showed that all indicators were met. This study conducted an internal consistency test on the four dimensions and the total scale of the Chinese version of C-CEP. The Cronbach’s *α* of the four dimensions were 0.852, 0.886, 0.895, and 0.853 respectively, and the Cronbach’s *α* of the total scale was 0.933. The retest reliability was 0.754 and the split-half reliability was 0.883. The four-factor structure explained a cumulative 74.897% of the total variance. All the content validity was met. The model-fit indicators were all within acceptable limits.

**Conclusion:**

The Chinese version of the Self-assessment scale for the community- based and emergency practice had good reliability and validity and could effectively assess medical students’ self-practice skills. The Chinese version of the C-CEP is a reliable and valid tool for assessing medical students’ community and emergency practice self-efficacy. It can explore the direction of community practice education for medical students and improve their practical emergency response capabilities.

## 1 Introduction

As a result of population aging, the age structure of China’s population is changing from “adult” to “old age,” and this rapid change in age structure poses a serious challenge to health care services and long-term care service models (1, 2). In the process of rapid demographic transition and economic and social development changes, the needs of older people are increasingly diversified, evolving from purely individual or family needs to social needs (3). The traditional sense of family older population care services is weakened and the combination of community-based primary health care services and older population care services is more in line with the current characteristics of older people’s needs (4, 5). In the Chinese context, community primary health care services for older people population still suffer from a lack of precision in service content, an inadequate service system, uneven and insufficient development, and limited-grassroots service capacity and information technology (6–8). Community primary health care services and long-term care services for older people population are not yet well developed in urban areas, while most rural areas have not yet established relevant long-term care and health care services (9, 10). Shortages of medical resources and low levels of care are not uncommon. The aging population has exacerbated the twin shortages of resources for medical healthcare and older population services, putting demands on improving the allocation and utilization of social resources. Human resources, the first resource of healthcare resources, are also key to improving medical primary healthcare services (11).

With the transformation of the medical model and the rapid development of the economy, people’s understanding and demands for their own health levels, as well as the requirements for doctors’ comprehensive clinical capabilities, are all increasing day by day (12). Medical practice is increasingly emphasizing respect for people and life. Medical education aims to cultivate doctors who promote the health of all mankind and is an important way for the country to input medical and health talent training to communities and grassroots areas. Medical schools are constantly defining the skills and knowledge that students need to ensure that graduates are properly prepared for the workplace (13, 14). The key point lies in cultivating the clinical practical operation ability of medical students, providing a guarantee for training the talents needed in primary health care. This comprehensive ability is cultivated and enhanced during the school years of medical students, which directly affects the effectiveness of their future medical activities (15).

The provision of planned community medical healthcare services is an ideal way to improve the capacity and effectiveness of primary care physician services, as well as the basis for implementing universal health coverage (16, 17). In current deep-aged China, the challenges of primary health care in the face of an aging population require medical students to constantly update their professional competencies and play an effective and important role in community clinical and acute care practice (18). Therefore, it is essential to find a suitable tool to allow medical students to assess their personal competencies in the course of their emergency practice in the community and clinic. The Self-assessment scale for the community- based and emergency practice (C-CEP), developed by Takao et al., was used as a simple and valid instrument to measure components of medical students’ attitudes, skills, and knowledge in community clinical and emergency practice (19). The scale had shown good reliability and validity in the Japanese population and could be used to measure medical students’ individual competencies in community clinical and emergency events.

The purpose of this study was to translate and cross-culturally adapt the Self-assessment scale for the community- based and emergency practice to form an appropriate self-assessment tool for Chinese medical students in community practice. The reliability and validity of its Chinese version were checked to allow for more validation and replication.

## 2 Methods

### 2.1 Study design

This study was conducted from January to March 2023 and was a cross-sectional study of this study was conducted from January to March 2023 and was a cross-sectional study of medical students using Self-assessment scale for the community- based and emergency practice. Inclusion criteria were ull-time fifth-year undergraduate medical students; had participated in clinical practice and apprenticeship; gave informed consent and volunteered to participate in this study; and were able to understand and complete the questionnaire independently. Exclusion criteria included foreign medical students; those who were unable to complete the survey for various reasons during the survey. The sample size was determined according to the general procedures of factor analysis. To ensure the accuracy of the factor analysis, a minimum of 10 study participants were asked to respond to each item (20). A total of 600 medical students were eventually recruited to participate in the study using a convenience sampling method.

### 2.2 Instruments

#### 2.2.1 General information questionnaire

After a thorough review of the literature by the research team, a general information questionnaire was developed, covering six areas: gender, age, ethnicity, city, being an only child and place of residence.

#### 2.2.2 Self-assessment scale for the community- based and emergency practice

The Self-assessment scale for the community- based and emergency practice (C-CEP), developed by Professor Wakabayashi et al. (19), was used to assess medical students’ self-competence in community and clinical emergency practice. The C-CEP consists of four dimensions: attitude and communication in emergency care, basic clinical skills, knowledge of community healthcare and knowledge of evidence-based medicine which were designed to allow medical students to measure and judge themselves on all four dimensions. The 15 items were scored on a five-point Likert scale from ‘strongly disagree’ to ‘strongly agree’ on a scale of 1–5, all of which are positive. The higher the score, the better the personal competence of student in dealing with acute and critical incidents in the community and clinical practice.

### 2.3 Procedure

#### 2.3.1 Cross-cultural adaptation of the scale

After obtaining authorization from the original scale development team, Professor Wakabayashi, the research team followed the Brislin translation model to translate and back-translate the scale (21). First, the original scale was independently translated by two researchers with proficient English skills, and the research team compared and synthesized the two to form a forward-translated version. Then this was reverse-translated by two foreign English teachers who had not been exposed to the original scale. The research team compared it with the source scale and discussed the differences between the different versions, resulting in a revised Chinese version of the Self-assessment scale for the community- based and emergency practice. Experts in medical education, community nursing, and other related fields were invited to further adapt the translated scale. A pre-survey of 30 medical students was conducted using the translated scale. During the survey, the study participants were asked whether they understood the content of the scale and whether they were clear about how to respond. The scale was further revised and refined based on the results of the pre-survey to determine the final version of the Chinese version of the Self-assessment scale for the community- based and emergency practice.

#### 2.3.2 Data collection process

After unified training, the research team went to two medical colleges and two hospitals of the same level in Jinzhou City, Liaoning Province and Nantong City, Jiangsu Province to investigate the medical students who met the inclusion criteria. The purpose and significance of the study were explained to participants prior to the survey and the anonymity, voluntary nature, and confidentiality of the survey were emphasized. With the help of school teachers, study subjects were placed in a quiet classroom to fill out questionnaires anonymously. Using convenience sampling, 632 study participants were recruited to participate in the study and the questionnaires were returned on the spot upon completion. Thirty-two invalid questionnaires were excluded and 600 valid data were collected. After 2 weeks, 30 medical students were asked to complete the Chinese version of the C-CEP again as a way to assess the retest reliability of the questionnaire.

### 2.4 Data analysis

Statistical description of the data, item analysis, reliability analysis and exploratory factor analysis were performed using SPSS 26.0 software. AMOS 26.0 software was used to perform validation factor analysis. *p* < 0.05 was considered statistically significant.

#### 2.4.1 Item analysis

The degree of discrimination of each item on the scale was judged according to the critical ratio (CR). The Chinese version of the C-CEP score was ranked in the order of high and low, with total scores above 61 (the former 27%) included in the high group and total scores below 50 (the latter 27%) included in the low group, and the difference between the two groups was calculated in terms of the mean score on each item. CR values > 3 surfaced the item with a good degree of distinction (22). A correlation analysis was conducted between the scores of each item and the total score, and items with a low correlation (*r* < 0.4) were removed (23). The Cronbach’s alpha coefficients of the deleted items did not exceed the Cronbach’s alpha coefficients of the original scale and were retained, while the opposite was deleted.

#### 2.4.2 Reliability analysis

The reliability of the Chinese version of the C-CEP was measured by internal consistency, split-half reliability, and retest reliability. The questionnaire items were divided sequentially into two halves and the correlation coefficient between the scores of the two halves was calculated to assess the split-half reliability of the whole scale. Two weeks later, 30 medical students were asked to re-answer the translated scale and correlation coefficients were calculated as a means of assessing its stability across time.

#### 2.4.3 Validity analysis

##### 2.4.3.1 Content validity

Seven experts in the relevant fields were invited to evaluate the content validity of the scale. The content validity index (I-CVI) for each item and the mean content validity index (S-CVI) for the scale were calculated based on the experts’ scores of the relevance of the items of the scale. I-CVI ≥ 0.78 and S-CVI ≥ 0.9 were considered as good content validity of the scale (24).

##### 2.4.3.2 Construct validity

The sample of 600 cases was randomly divided into two groups (*n* = 300) for exploratory factor analysis and confirmatory factor analysis, respectively. The Kaiser-Meyer-Olkin value (KMO) and Barlett’s spherical test results were used to determine whether the scale data were suitable for EFA. Normally, the scale with KMO values > 0.6 and Bartlett’s spherical test *χ*^2^ values reaching significant differences (*p* < 0.05) could be subjected to exploratory factor analysis (25). Principal component analysis and maximal rotation of variance were used to calculate the accumulation of factor loadings and their contribution rates, to determine whether the Chinese version of the scale was reasonable in terms of entry setting and dimension classification. A structural equation model (SEM) was constructed using AMOS 26.0 software to plot the items and dimensions. The CFA used the chi-square degree of freedom (*χ*^2^/df), the root mean square error of approximation (RMSEA), the goodness-of-fit index (GFI), the comparative fit index (CFI) and the incremental fit index (IFI) to assess the overall structural fit of the scale.

## 3 Results

### 3.1 General information

A total of 600 study participants were included in this study, 226 (37.7%) males and 374 (62.3%) females with a mean age of 23.06 ± 1.26 years. Over half of the study subjects were only children and other demographic information is detailed in Table 1.

**Table 1:** General demography data (*n* = 600).

Characteristics	Group/Mean±SD	*n*	%
Age	23.06 ± 1.26		
Sex	Male	226	37.7
	Female	374	62.3
Ethnicity	Han Chinese	390	65.0
	Other ethnic groups	210	35.0
City	Jinzhou	402	67.0
	ShenYang	198	33.0
Place of residence	Urban	367	61.2
	Rural	233	38.8
Only child	Yes	449	74.8
	No	151	25.2

### 3.2 Item analysis

The critical ratio and correlation coefficient were used to evaluate the discrimination and homogeneity of the Chinese version of the C-CEP items. The results showed that the CR values for all items ranged from 15.036 to 20.052, all >3 and *p* < 0.05. The correlation coefficient *r* between the items and the total score ranged from 0.642 to 0.764, all > 0.4 and statistically significant (*p* < 0.05). After deleting the items, the results of the Cronbach’s alpha for the scale ranged from 0.927 to 0.931, none of which exceeded the Cronbach’s alpha for the scale (0.933). The results showed that all items of the Chinese version of the C-CEP were retained (Table 2).

**Table 2:** Item analysis for Chinese version of the C-CEP.

Item	Critical ratio	Correlation coefficient between item and total score	Cronbach’s Alpha if item delete
1	15.036	0.642	0.931
2	16.532	0.671	0.930
3	15.868	0.652	0.931
4	17.810	0.736	0.928
5	16.509	0.691	0.930
6	19.136	0.748	0.928
7	20.153	0.746	0.928
8	17.668	0.722	0.929
9	18.575	0.727	0.929
10	20.502	0.735	0.928
11	19.739	0.748	0.928
12	20.349	0.764	0.927
13	17.856	0.732	0.929
14	17.439	0.711	0.929
15	19.138	0.753	0.928

### 3.3 Reliability analysis

The reliability of the Self-assessment scale for the community- based and emergency practice was 0.933, with Cronbach’s alpha coefficients ranging from 0.852 to 0.895 for the four dimensions. The scale had a split-half reliability of 0.833 and retest reliability of 0.754.

### 3.4 Validity analysis

#### 3.4.1 Content validity

The content validity of the scale was assessed using a 4-point Likert scale, with items rated by experts as “1” or “2” was considered “not relevant” and items rated as “3” or “4” was considered “relevant.” The results showed that the I-CVI of the Chinese version of the C-CEP items ranged from 0.857 to 1, and the S-CVI was 0.924.

#### 3.4.2 Construct validity

**Figure 1:**
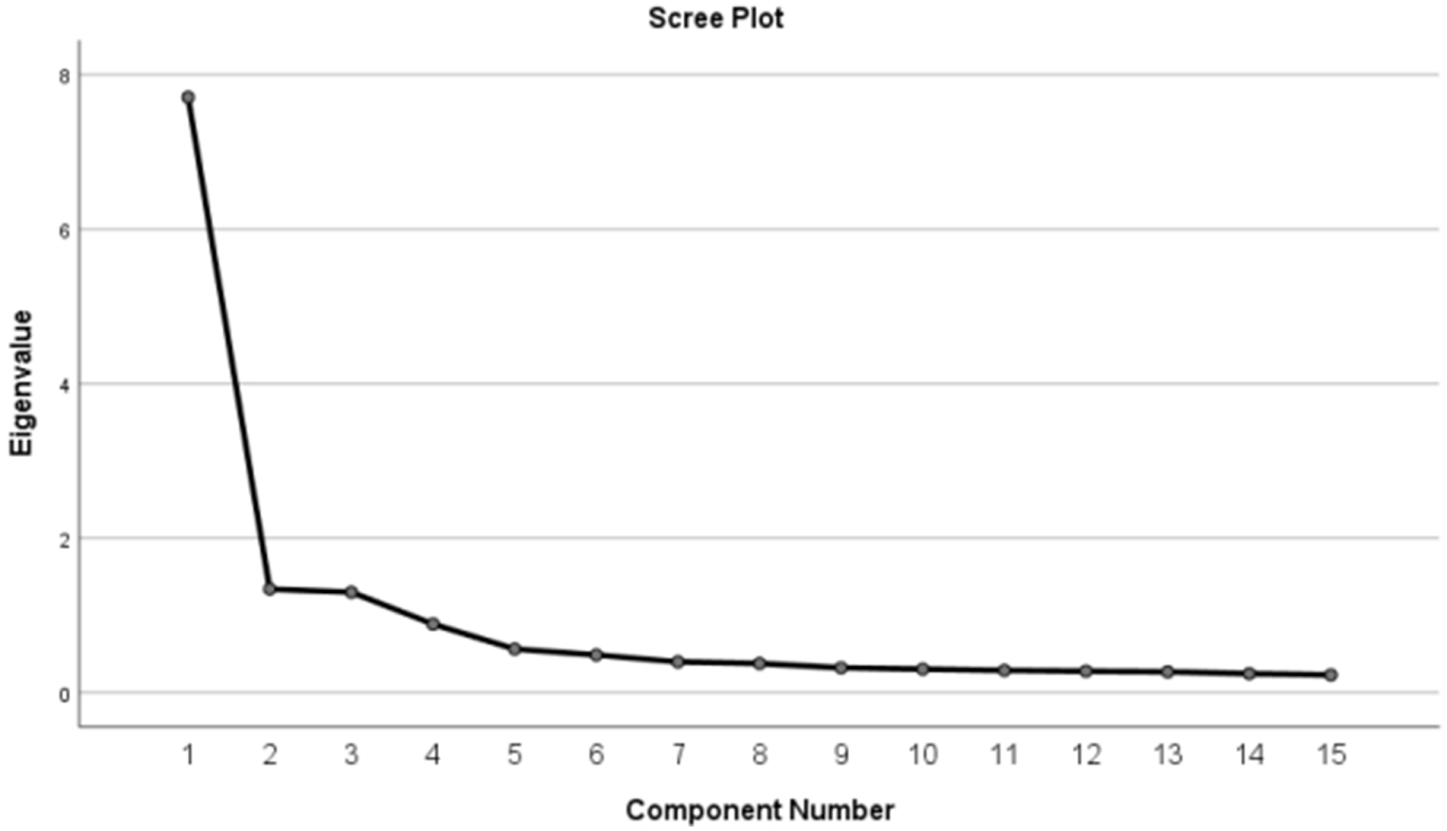
Screen plot of exploratory factor analysis for Chinese version of the C-CEP.

A total of 15 items were included in the exploratory factor analysis. The KMO value for the scale was 0.929 and the Barlett’s test of sphericity *χ*^2^ = 2855.423 (*p* < 0.001), allowing for EFA. A total of 4 factors were extracted by orthogonal rotation of the scale using principal component analysis and maximum variance. Factor loadings are shown in Table 3. 74.897% of the variance was explained cumulatively by the 4-factor model. Factor naming was based on the original scale. The scree plot is detailed in Figure 1.

**Table 3:** Factor loadings of exploratory factor analysis for Chinese version of the C-CEP.

Item	Factor 1	Factor 2	Factor 3	Factor 4
Communication among medical team in ER	-	-	0.855	-
Communication to patient in ER	-	-	0.828	-
Communication to staffs of other hospitals	-	-	0.782	-
Explaining to patients, to obtain consent of examinations	-	0.735	-	-
The methods and indications of basic procedures	-	0.822	-	-
The meanings of vital signs in ER	-	0.803	-	-
Physical examinations	-	0.752	-	-
The roles of the primary-care physician at the institution	0.678	-	-	-
The characteristics of the community’s health problems	0.772	-	-	-
The difference in primary physician’s roles according to communities	0.808	-	-	-
The role of a primary-care physician in the emergency setting	0.783	-		-
The role of medical insurance in the emergency room	0.709	-	-	-
Referring to the evidence, to solve clinical problems	-	-	-	0.663
Explaining the management for common diseases, using evidence	-	-	-	0.832
The composition of medical term in the emergency room	-	-	-	0.735

**Figure 2:**
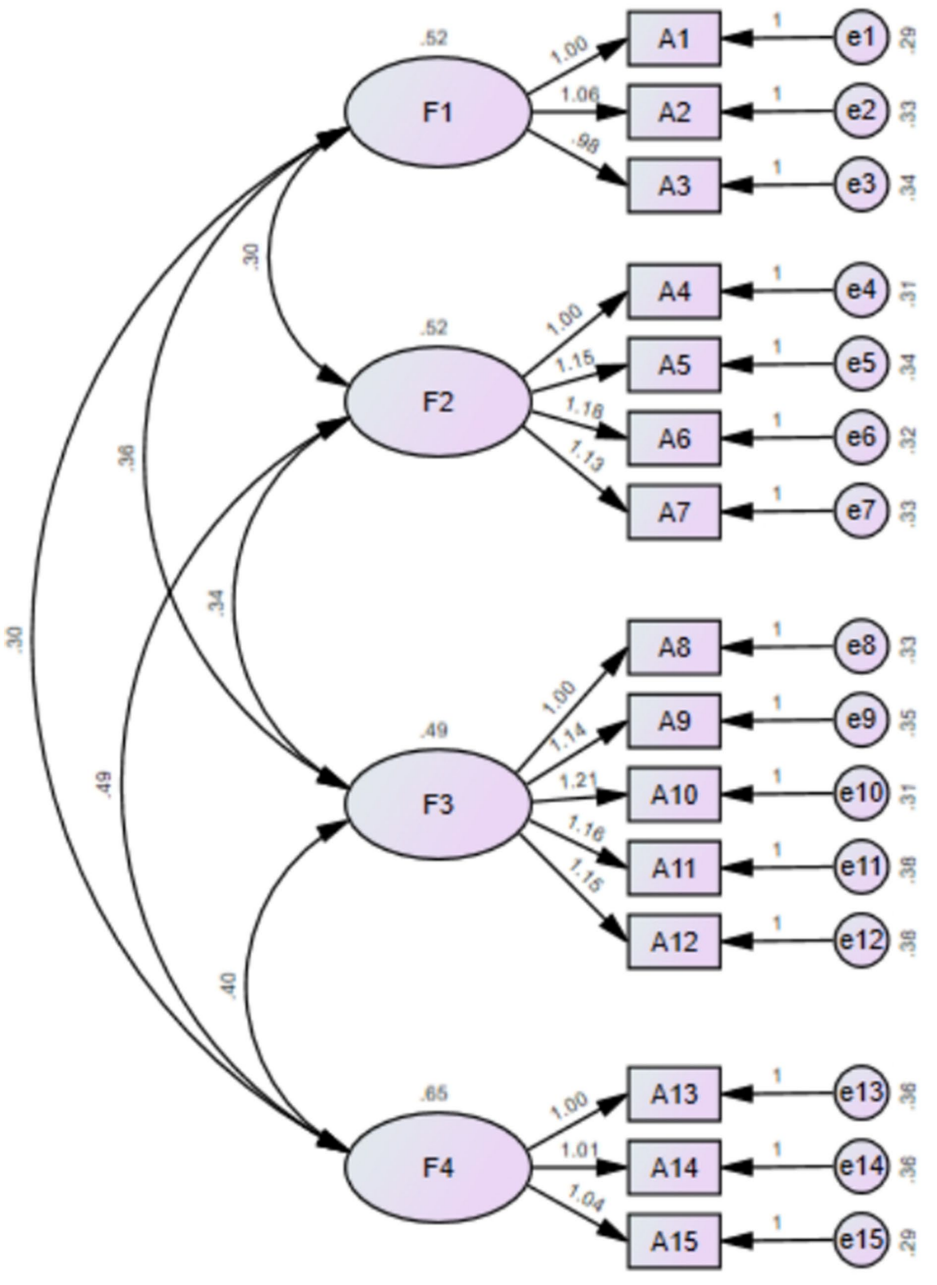
Standardized four-factor structural model of the C-CEP.

In the confirmatory factor analysis, the great likelihood method was used to analyze the goodness of fit between the items and the dimensions. The results showed that *χ*^2^/df was 1.906 < 3 and RMSEA was 0.055 < 0.08; IFI was 0.974 > 0.9; TLI was 0.967 > 0.9; CFI was 0.973 > 0.9; PGFI was 0.51 > 0.5; and PNFI was 0.757 > 0.5. This demonstrated that the factor model of the translated scale was consistent with the envisaged structure (26) (Figure 2).

## 4 Discussion

### 4.1 The content attributes of the Chinese version of the self-assessment scale for the community- based and emergency practice

In order to make the scale items more consistent with Chinese language expressions and cultural background, the study was translated and back-translated in strict accordance with the Brislin translation principles to ensure linguistic equivalence (21). The cross-cultural adaptation was carried out through Delphi expert consultation and pre-survey. During the cross-cultural adaptation process, uncertain topics were discussed and agreed upon by the research team several times to ensure consistency between the Chinese version of the scale and the content of the source scale. Based on the feedback from the participants in the pre-survey, the scale was further revised and adjusted to ensure the clarity and comprehensibility of the Chinese version of the C-CEP, resulting in the final version of the Chinese version of the Self-assessment scale for the community- based and emergency practice. The scale contains 4 dimensions with 15 items and was easy to read, suitable for medical students to conduct self-assessment and could be widely used in clinical medical research and medical education evaluation.

### 4.2 The measurement properties of the Chinese version of the self-assessment scale for the community- based and emergency practice

Item analysis aimed to determine the reliability of each item of the scale so that the items could be screened or modified as a way to verify the appropriateness of the item in this scale (22). The critical ratio and correlation coefficient methods were used in this study to conduct item analyzes for the Chinese version of the Self-assessment scale for the community-based and emergency practice. The critical ratio method showed that there was a significant difference in CR values between the high and low subgroups for comparison on each item (CR > 3) (27). This indicated that the scale was well differentiated and could be used to measure medical students’ individual practice in community and clinical emergency care. The results of the correlation coefficient method showed that all items had a correlation coefficient *r* > 0.4 with the total scale, indicating good homogeneity between the scale items. Therefore, all items of the Chinese version of the Self-assessment scale for the community- based and emergency practice were retained.

Reliability, which reflects the consistency and stability of the measurement results, determines whether the instrument can consistently measure the things and variables to be evaluated (28). Reliability is divided into internal reliability and external reliability. Internal reliability is often expressed in terms of Cronbach’s alpha coefficient and split-half reliability, with Cronbach’s alpha coefficient > 0.8 for the total scale and Cronbach’s alpha coefficient > 0.6 for each dimension being considered to have good internal reliability. In this study, the overall Cronbach ‘*α* coefficient of the scale was 0.933 and the range of Cronbach ‘*α* coefficients for each dimension was 0.852 to 0.895, all greater than 0.80. This suggested that the Chinese version of the scale had ideal internal consistency and that the dimensions were closely related to the scale as a whole (29). The Chinese version of the C-CEP had a split-half reliability of 0.883, further indicating the good correlation and stability of the items of the research instrument. Retest reliability is a test of the external reliability of a scale. The retest reliability value in this study was 0.754, indicating that the scale had good stability across time (30).

Validity refers to the extent to which the measured results reflect what is intended to be examined, and the more closely the results match what is to be examined, the higher the validity. This study measured both content validity and structural validity. Content validity responds to whether the research content measured by the scale is consistent with the purpose of the study. The relevance was evaluated by experts in the relevant field. The CVI was calculated based on the number of experts scoring the scale, and the closer the value to 1, the better the content validity of the scale. The results of the study showed that the Chinese version of the C-CEP had the I-CVI of 0.857 ~ 1 and the S-CVI of 0.924, both of which were greater than the reference values, indicating that the scale had good representation and coverage of the evaluation content and good content validity (31). Structural validity reflects the degree of correspondence between the actual situation of the scale and the theoretical concepts, of which factor analysis is the most commonly used method. In this study, the structural validity of the scales was evaluated by exploratory factor analysis and confirmatory factor analysis. In exploratory factor analysis, the scale was considered to have good structural validity when the factor composition was the same as the structure of the original scale, the cumulative variance contribution of the factors exceeded 50% and all items loaded greater than 0.4 on their corresponding factors, while loading on their factors was less than 0.4 (32). The results show that the Chinese version of the C-CEP had KMO values >0.6 and *χ*^2^values reached significant differences (*p* < 0.05) in Bartlett’s spherical test, indicating that it was suitable for exploratory factor analysis (25). The 4-factor model cumulatively explained 74.897% of the variance, indicating that the scale had good construct validity. The results of the confirmatory factor analysis showed that *χ*^2^/df < 2, RMR < 0.08, CFI, IFI, NFI and RFI were all greater than 0.90, which met the fit criteria, suggesting that the relationship between the items and dimensions in the scale fit well with the theoretical model (26).

### 4.3 The practicality of the Chinese version of the self-assessment scale for the community- based and emergency practice

With the increasing aging of the population and the rising incidence of chronic diseases, there has been a gradual transition in the medical health service model from acute care facilities to community care facilities (33, 34). Community medical health care, based on social, group and individual service needs, plays an important role in primary medical health care. In order to provide long-term, continuous, high-quality medical healthcare services to the community, it is important to focus on building the primary medical workforce, developing the problem-solving skills of medical students in dealing with community and clinical emergencies, and promoting the high-quality development of primary medical services (35, 36). In the Chinese context, there is a lack of research tools to assess medical students’ competencies related to community and clinical practice. The Chinese version of Self-assessment scale for the community-based and emergency practice focused on the field of community medicine and was used to assess aspects of medical students’ attitudes, skills and knowledge during emergency care. The introduction of the scale provided a quantitative tool for evaluating the individual competencies of medical students in practice in China, as well as a basis for medical educators to adopt effective measures to enhance community and clinical practice, thus prompting more medical students to choose to work in social de-medicine to maintain and promote the health of the community population. In the questionnaire survey, medical students all indicated that the Chinese version of the C-CEP items were easy to understand and accept, and the acceptance and completion rates were more satisfactory, indicating that the Chinese version of the C-CEP is more feasible.

### 4.4 Limitations

Due to the reasons of time and conditions, this study adopted the method of convenience sampling and only selected medical students from two urban medical colleges. The degree of bias was high, which might have affected the representativeness of the sample to a certain extent. The further research plan in the future is to conduct a survey among medical students in medical colleges and universities in different regions, further develop and improve the scale, in order to understand the cognitive situation of medical students.

## 5 Conclusion

This study was cross-culturally debugged and tested for validity to develop Self-assessment scale for the community- based and emergency practice (C-CEP) appropriate for Chinese medical students. The scale consisted of 4 dimensions with 15 items and showed satisfactory reliability and validity. The Chinese version of the C-CEP provides a more comprehensive measure of medical students’ perceptions and competencies in community medicine and primary health care and can be used as a tool to assess the effectiveness of the curriculum and provide a basis for medical educators to provide targeted professional education in community medicine.

## Data Availability

The raw data supporting the conclusions of this article will be made available by the authors, without undue reservation.
